# Adaptive evolution of plasmid and chromosome contributes to the fitness of a *bla*_NDM_-bearing cointegrate plasmid in *Escherichia coli*

**DOI:** 10.1093/ismejo/wrae037

**Published:** 2024-03-04

**Authors:** Ziyi Liu, Yanyun Gao, Mianzhi Wang, Yuan Liu, Fulin Wang, Jing Shi, Zhiqiang Wang, Ruichao Li

**Affiliations:** College of Veterinary Medicine, Yangzhou University, Yangzhou, 225009 Jiangsu Province, People's Republic of China; Jiangsu Co-innovation Center for Prevention and Control of Important Animal Infectious Diseases and Zoonoses, Jiangsu Key Lab of Zoonosis, Yangzhou, 225009 Jiangsu Province, People's Republic of China; Institute of Comparative Medicine, Yangzhou University, Yangzhou, 225009 Jiangsu Province, People's Republic of China; College of Animal Science and Technology & College of Veterinary medicine, Zhejiang Agriculture and Forestry University, Hangzhou, 311300 Zhejiang Province, People's Republic of China; College of Veterinary Medicine, Yangzhou University, Yangzhou, 225009 Jiangsu Province, People's Republic of China; Jiangsu Co-innovation Center for Prevention and Control of Important Animal Infectious Diseases and Zoonoses, Jiangsu Key Lab of Zoonosis, Yangzhou, 225009 Jiangsu Province, People's Republic of China; College of Veterinary Medicine, Yangzhou University, Yangzhou, 225009 Jiangsu Province, People's Republic of China; Jiangsu Co-innovation Center for Prevention and Control of Important Animal Infectious Diseases and Zoonoses, Jiangsu Key Lab of Zoonosis, Yangzhou, 225009 Jiangsu Province, People's Republic of China; College of Veterinary Medicine, Yangzhou University, Yangzhou, 225009 Jiangsu Province, People's Republic of China; Jiangsu Co-innovation Center for Prevention and Control of Important Animal Infectious Diseases and Zoonoses, Jiangsu Key Lab of Zoonosis, Yangzhou, 225009 Jiangsu Province, People's Republic of China; Institute of Comparative Medicine, Yangzhou University, Yangzhou, 225009 Jiangsu Province, People's Republic of China; Department of Pathogen Biology, School of Medicine and Holistic Integrative Medicine, Nanjing University of Chinese Medicine, Nanjing, 210023 Jiangsu Province, People's Republic of China; Department of Pathogen Biology, School of Medicine and Holistic Integrative Medicine, Nanjing University of Chinese Medicine, Nanjing, 210023 Jiangsu Province, People's Republic of China; College of Veterinary Medicine, Yangzhou University, Yangzhou, 225009 Jiangsu Province, People's Republic of China; Jiangsu Co-innovation Center for Prevention and Control of Important Animal Infectious Diseases and Zoonoses, Jiangsu Key Lab of Zoonosis, Yangzhou, 225009 Jiangsu Province, People's Republic of China; Institute of Comparative Medicine, Yangzhou University, Yangzhou, 225009 Jiangsu Province, People's Republic of China; Institute of Agricultural Science and Technology Development, Yangzhou, 225009 Jiangsu Province, People's Republic of China; College of Veterinary Medicine, Yangzhou University, Yangzhou, 225009 Jiangsu Province, People's Republic of China; Jiangsu Co-innovation Center for Prevention and Control of Important Animal Infectious Diseases and Zoonoses, Jiangsu Key Lab of Zoonosis, Yangzhou, 225009 Jiangsu Province, People's Republic of China; Institute of Comparative Medicine, Yangzhou University, Yangzhou, 225009 Jiangsu Province, People's Republic of China

**Keywords:** blaNDM, cointegrate plasmid, E. coli, fitness cost, adaptive evolution

## Abstract

Large cointegrate plasmids recruit genetic features of their parental plasmids and serve as important vectors in the spread of antibiotic resistance. They are now frequently found in clinical settings, raising the issue of how to limit their further transmission. Here, we conducted evolutionary research of a large *bla*_NDM_-positive cointegrate within *Escherichia coli* C600, and discovered that adaptive evolution of chromosome and plasmid jointly improved bacterial fitness, which was manifested as enhanced survival ability for *in vivo* and *in vitro* pairwise competition, biofilm formation, and gut colonization ability. From the plasmid aspect, large-scale DNA fragment loss is observed in an evolved clone. Although the evolved plasmid imposes a negligible fitness cost on host bacteria, its conjugation frequency is greatly reduced, and the deficiency of anti-SOS gene *psiB* is found responsible for the impaired horizontal transferability rather than the reduced fitness cost. These findings unveil an evolutionary strategy in which the plasmid horizontal transferability and fitness cost are balanced. From the chromosome perspective, all evolved clones exhibit parallel mutations in the transcriptional regulatory stringent starvation Protein A gene *sspA*. Through a *sspA* knockout mutant, transcriptome analysis, *in vitro* transcriptional activity assay, RT-qPCR, motility test, and scanning electron microscopy techniques, we demonstrated that the mutation in *sspA* reduces its transcriptional inhibitory capacity, thereby improving bacterial fitness, biofilm formation ability, and gut colonization ability by promoting bacterial flagella synthesis. These findings expand our knowledge of how cointegrate plasmids adapt to new bacterial hosts.

## Introduction

Bacterial plasmids play a significant role in facilitating horizontal gene transfer and harbor a diverse array of accessory genetic elements that are crucial to the adaptation of bacterial species [1]. Cointegrate plasmids, as a representative type of plasmids, contain the genetic information of two or more original plasmids and are characterized by a high abundance of antimicrobial resistance (AMR) genes and mobile elements [2-4]. Their formation is usually mediated by the role of insertion sequences and recombination between homologous regions in diverse versatile plasmids. Currently, numerous studies have reported the presence of cointegrate plasmids in clinical isolates [5, 6] or transconjugants [7, 8], thereby posing a potential threat to public health. Despite their increasing abundance, these plasmids exhibit uneven distribution across prokaryotic taxa, implying that they may confer varying levels of potential burden to different species [9].

In addition to some plasmid–chromosome pairs within bacteria that are ecologically compatible [10, 11], the acquisition of an external plasmid is generally associated with a fitness cost for the host bacteria [12], which may ultimately lead to the extinction of plasmid. Despite the well-known fitness costs, plasmids are ubiquitous and seem to spread without any obstacles, which is referred to as the “plasmid paradox” [13]. The widespread prevalence of plasmids in bacterial genomes poses a challenging conundrum, and solving the plasmid paradox remains an active area of research [14]. Recently, multiple solutions to the plasmid paradox have been proposed, including understanding the plasmid fitness costs as well as exploring compensatory evolution that reduces the burden of plasmid carriage [15]. An increasing number of studies have focused on the evolutionary dynamics and fates of plasmid-carrying bacteria to elucidate how compensation evolution mitigates the cost of plasmids. For instance, chromosomal gene mutations and specific regions deletions could facilitate the persistence of multidrug-resistant IncHI2 plasmids in *Salmonella* Typhimurium [16]. In an *Escherichia coli* strain MG1655 carrying the multidrug-resistant plasmid RP4, chromosomal mutations and transcriptional modifications facilitated phenotypic changes, thereby enhancing colonization ability and plasmid transferability [17]. Because these identified critical changes are strongly associated with improved bacterial fitness, the investigation of the molecular mechanisms underlying bacterial adaptive evolution is crucial in mitigating the dissemination of multidrug-resistant plasmids.

In a previous study, we identified pL53T as a cointegrate plasmid resulting from the homologous recombination of two IS*26* elements on an IncX3 plasmid and a multidrug-resistant IncFII plasmid, and it carried AMR genes, including *fosA3*, *bla*_NDM-5_, *bla*_CTX-M-55_, and *bla*_TEM-1B_, which poses a significant threat to public health [18]. Given the widespread prevalence of IncX3 and IncFII plasmids in *E. coli* [19, 20], the convergence of such MDR plasmids pose a significant concern. To learn the evolution and underlying molecular mechanisms of the cointegrate plasmid, we introduced the plasmid into *E. coli* C600 and conducted a long-term evolution experiment under selection pressure to decipher the molecular mechanisms driving the adaptative evolution. Our findings reveal the role of *psiB* in facilitating horizontal transferability of the cointegrate plasmid. In addition, the *sspA* mutation in the chromosome enhances bacterial fitness, biofilm formation, and gut colonization ability. This adaptive evolutionary strategy broadens our understanding of plasmid adaptation to bacterial hosts in different ecological niches and enables us to identify critical targets for curtailing plasmid transmission.

## Materials and methods

### Bacterial strains, plasmids, and culture conditions

The *E. coli* C600 strain, a prototypical K-12-derived laboratory strain, is widely recognized as an optimal material for conducting molecular microbiology and bacterial physiology research [21]. The cointegrate plasmid pL53T was introduced into the C600 via electroporation to construct ancestral strain C600-pL53T. A detailed description of pL53T can be found in the results section. Unless otherwise specified, all bacteria were cultured in LB broth at 37°C with shaking at 200 rpm by default.

### Experimental evolution and plasmid stability measurement

In the initial stage of the experiment, the ancestral strain C600-pL53T was serially passaged in triplicate under antibiotic-free LB broth every day (twice a day) for 30 days (60 × log_2_ 1000) to achieve ~600 generations calculated as previously described [16]. The detailed process is as follows: 5 μl of bacterial cultures were inoculated into 5-ml fresh LB broth every 12 h. The bacterial cultures were stored every 5 days. To evaluate the plasmid stability, the stored cultures were streaked onto antibiotic-free LB agar plates and randomly selected 96 single colonies on each plate. PCR targeting at IncX3 replication gene and two fusion sites (Table S1) was applied to confirm the presence of the plasmid and calculate the proportion of plasmid-carrying bacteria in each generation. The bacterial cultures preserved in this step can be used as a control group for subsequent evolutionary experiments.

Given that plasmid pL53T was not stable in C600 under antibiotic-free conditions, we therefore subjected the ancestral strain C600-pL53T to serial passages in LB broth containing meropenem (2 μg/ml) and fosfomycin (16 μg/ml) for 30 days in triplicate, among which the concentration of antibiotics was the subinhibitory concentration determined based on MIC results. The detailed procedure is the same as above. After the passage, the bacterial cultures were streaked onto the antibiotic-free LB agar plate. One single colony was randomly selected from each of the three parallel groups, and a total of three evolved clones were used for further research. The plasmid stability procedure for the evolved clones remains unchanged, excepted for the reduction in the passage of 15th day.

### Competition assays

We selected the ancestral strain, three evolved strains, C600ΔsspA-pL53T, and C600-pL53TΔpsiB to determine the improvement in fitness during adaptative evolution and the impact of gene deletion on plasmid fitness cost. The following methods were employed for the bacteria competition assay: cultures of the tested strains and the ancestral plasmid-free strain were diluted to a 0.5 McFarland standard and mixed at a ratio of 1:1 in 5-ml antibiotic-free LB broth. The mixtures were then incubated at 37°C for 24 h, followed by appropriate dilution and plating onto LB agar with or without antibiotics (2 mg/l of meropenem and 16 mg/l of fosfomycin). The number of colonies of the tested strains and the ancestral plasmid-free strain was counted at both 0 and 24 h. The relative fitness (RF) of the tested strains was determined by calculating the ratio of Malthusian parameters [22], as follows: RF = ln(NRt/NRi)/ln(NSt/NSi), where NRt represents the number of resistant clones at 24 h, NRi represents the number of resistant clones at the initial time point, NSi represents the number of susceptible clones at the initial time point, and NSt represents the number of susceptible clones at 24 h. An RF value lower than 1 indicates the presence of fitness cost, or vice versa.

### Measurement of biofilm formation

The biofilm formation abilities of the tested strains were evaluated according to previously described methods [2]. Briefly, overnight cultures were diluted to an approximate 0.5 MacFarland standard, and 2 μl of dilutions was pipetted into 96-well plates containing 198-μl LB broth. After incubation at 37°C for 48 h, the cultures were discarded and the wells were washed twice with PBS to remove the planktonic bacteria. Subsequently, the biofilms were stained with a 0.1% crystal violet solution for 10 min and rinsed with PBS. After air-drying for 30 min, each well was treated with 30% formic acid to dissolve the biofilms. The biofilm yield was quantified by measuring absorbance at 590 nm.

### Determination of plasmid conjugation frequency

Strain C600-pL53T, C600e-pL53T-1e, C600e-pL53T-2e, C600-pL53T-3e, and C600-pL53TΔpsiB were utilized as donor strains for plasmid conjugation frequency measurement, with *E. coli* J53 (resistant to sodium azide) or J53::psiB serving as the recipient strain. The plasmid conjugation frequency was calculated by solid mating [23]. After both the donor and recipient cultures reached an optical density of 0.8 at 600 nm, equal volumes (500 μl) of each culture were mixed. The mixed cultures were centrifuged at 3000*g* for 5 min, and the supernatants were discarded. The cell pellets were then washed with an equal volume of LB medium and subsequently resuspended in 100 μl of LB before being dropped onto LB agar plates. These plates were incubated at a temperature of 37°C for a duration of 3 h. The number of transconjugants and recipient cells was determined by enumerating the colonies growing on plate containing sodium azide (200 mg/l) and meropenem (2 mg/l), and plate containing only sodium azide (200 mg/l), respectively. Conjugation frequencies were calculated as the ratio of transconjugants to recipient cells.

### Motility test

In order to investigate whether differential expression of flagella-related genes contributes to changes in bacterial motility, a motility assay was performed following established protocols with biological triplicates [24]. Soft MH agar plates (0.3%) were prepared and cultures adjusted to 0.6 McFarland by PBS were inoculated at the center of the semisolid medium. After incubating at 37°C for 72 h, the diameter of bacterial growth rings was measured.

### Quantitative real-time PCR

The messenger RNA expression was evaluated using RT-qPCR. Bacterial total RNA was extracted with the Bacteria RNA Extraction Kit (Vazyme Biotech Co., Ltd, China), followed by reverse transcription into cDNA by HiScriptR^R^III RT SuperMix for qPCR Kits (Vazyme Biotech Co., Ltd, China). The chromosomal marker 16S rRNA gene was used as the reference gene in qPCR, and the relative expression level was determined by the comparative ^C^_T_(^ΔΔC^_T_) method [25]. The primers used in this experiment are listed in Table S1.

### CRISPR system-based gene knockout and *psiB* gene complementation

A two-plasmid CRISPR/Cas9 system (Fig. S1) was utilized for *psiB* and *sspA* knockout, following the previously described protocol [26]. Initially, a donor DNA with two homologous arms was integrated into the sgRNA plasmid to generate an intermediate plasmid. Subsequently, a specific spacer (N20) was inserted between the *araB* promoter and the gRNA scaffold in the intermediate plasmid using single PCR and single Gibson Assembly. The plasmid pHCY-25A was transformed into chemically competent C600-pL53T cells to generate the strain C600-pL53T-pHCY-25A. The resulting strain C600-pL53T-pHCY-25A was then rendered chemically competent and subjected to transformation with 5 μl of sgRNA plasmid. The transformants were recovered from an LB plate supplemented with kanamycin, chloramphenicol, and glucose after incubation at 30°C for 12 h. The grown strain was induced with IPTG and L–arabinose before being spread onto an LB plate containing chloramphenicol, kanamycin, and L–arabinose. The primers listed in Table S1 were used to verify the knockout of targeted genes.

To complement the *psiB* gene, we first amplified a complete fragment of the *psiB* gene with its promoter using zeropsiB-F and zeropsiB-R primers (Table S1). Following purification of the product, a vector carrying this fragment was constructed using pBackZero-T Vector Cloning Kit (TaKaRa Biological Technology Co., LTD, China) and subsequently chemically transformed into *E. coli* J53 and verified by PCR.

### Whole-genome sequencing and bioinformatic analysis

The ancestral strain and three evolved clones underwent whole-genome sequencing (WGS). Genomic DNA was extracted using the FastPure Bacteria DNA Isolation Mini Kit (Vazyme, Biotech Co., Ltd, China) and sequenced via short-read sequencing (2 × 150 bp) with the HiSeq 2500 system (Illumina). The raw sequences were assembled using SPAdes [27], and contigs < 500 bp were excluded. The cointegrate plasmid sequence obtained in previous study [18] was compared with these assembled data to illustrate the alterations of plasmid structure by CLC Genomics Workbench 12. Single-intergenic nucleotide polymorphism (SNP) analysis was performed using Snippy (4.0.2) against the genome of the ancestral strain [28]. The effect of single-base mutations on protein function was predicted by Phyre2 [29], and the sequence profiles and mutations were automatically generated by Phyre Investigator tool.

### *In vitro* transcription assays

The impact of *sspA* gene mutation on RNA polymerase (RNAP) activity was investigated through *in vitro* transcription assays using the mango method [30]. The reaction system, consisting of 50-nM *gadA* promoter Mango DNA, 200-nM *E. coli* σ^70^-RNAP holoenzyme in a total of volume of 80 μl, was performed at 37°C in a reaction buffer containing 40-mM Tris–HCL, PH 8.0, 10-mM MgCl_2_, 0.5% (vol/vol) glycerol, 100-mM KCl, 1-mM DTT, and 0.1% Tween-20. The reaction was preincubated at 37°C for 5 min, followed by the addition of 10-μl SspA or its mutant protein (final concentration 0.312, 0.625, 2.5, and 5 μM), the NTP mix (0.1 mM; final concentration), and Tol-biotin (1 μM; final concentration) in order. The reaction system was incubated for 30 min, followed by measurement of fluorescence signals using a plate reader (SPARK, TECAN, Inc.) with excitation wavelength and emission wavelength of 510 and 535 nm, respectively. Further details about the preparation of SspA protein, σ^70^, and RNAP core enzyme can be found in the Supplementary Information.

### RNA-seq and bioinformatics

Strain C600ΔsspA-pL53T, C600-pL53T, and C600e-L53T-1e were cultured in LB broth until reaching the exponential phase. Total RNA was extracted from the samples using RNA isolator Total RNA Extraction Reagent (Vazyme Biotech Co., Ltd, China) and quantified with a NanoDrop spectrophotometer (Thermo Scientific, MA, USA). The HiSeq 2000 system (Illumina) was utilized to sequence the total RNA of these strains, with the read length as 2 × 10 (PE100) (Azenta, Suzhou, China). Following filtration, high-quality reads were mapped to the reference genome of C600-pL53T. Differentially expressed genes (DEGs) were estimated using RPKM (reads per kilobase per million reads) method, with a significance threshold of *P* values ≤ .05 and fold change (FC) values ≥ 2 (log2 FC ≥ 1 or log2 FC ≤ −1) [31]. The differences between the two treatments were analyzed by the cuffdiff program (http://cufflinks.cbcb.umd.edu/).

### Observation of bacterial morphology

To verify the transcriptome results, strain C600ΔsspA-pL53T, C600-pL53T, and C600e-L53T-1e were selected for morphological observation by scanning electron microscope. The tested strains were cultured overnight in LB broth, washed three times with PBS, and fixed with 2.5% glutaraldehyde (Solarbio, Beijing, China) at 4°C overnight. Subsequently, the fixed bacteria were dehydrated with a series of increasing alcohol concentrations (30%, 50%, 70%, 90%, and 100%). The dehydrated bacteria were then sequentially dried, adhered to a plated, and observed by GeminiSEM 300 (Carl Zeiss, Jena, Germany).

### *In vivo* animal experiments

#### Galleria mellonella larvae infection assay

According to the previous study, *Galleria mellonella* larvae infection assay was performed to estimate the virulence potential of the tested strains [32]. Briefly, larvae about 300 mg were resuscitated in a 37°C incubator for 4 h. Overnight cultures were washed and adjusted to 10^6^ CFU/ml using PBS. Ten healthy larvae were grouped and challenged with 10 μl of cultures by the Hamilton syringe of 25 μl, and the group injected with PBS was considered as the negative control. The groups were incubated in sterilized Perti dishes at 37°C for 3 days and the survival rate of each group was recorded daily.

#### In vivo competition assays

According to the previous study [17], we conducted a comparative analysis of the competitiveness between the ancestral strain and three evolved clones in mouse livers and spleens. The overnight cultures of the tested strains and the plasmid-free ancestral strain were diluted and mixed at a 1:1 ratio to obtain a 250-μl mixture of 10^9^ CFU/ml in PBS. Female BALB/c mice aged between 6 and 8 weeks were purchased from the Comparative Medicine Centre of Yangzhou University (Jiangsu, China), with three mice being challenged with bacteria in each group. These mixtures were intraperitoneally injected into mice. After 24 h of competition, the mice were anesthetized with isoflurane and subsequently euthanized. The spleen and liver were then aseptically extracted, weighed, and homogenized. The processed organs were further plated onto LB plates with or without the addition of antibiotic (rifampin: 200 mg/l, meropenem: 2 mg/l, and fosfomycin: 16 mg/l). The following day, the number of clones grown on each type of plate was enumerated and used to calculate an *in vivo* RF based on collected data.

#### Gut colonization assays

A total of 25 BALB/c female mice, aged 6–8 weeks, were procured from the Comparative Medicine Centre of Yangzhou University. The mice were allocated randomly into four groups (five per group) and subjected to pretreatment with streptomycin [33]. Briefly, the mice were initially fed with sterile food and drinking water supplemented with streptomycin sulfate (5 g/l) for 5 days, followed by a 5-day period of antibiotic-free sterile water consumption before bacterial colonization. At the end of this stage, fecal samples from these mice were collected and milled. The resulting material was then diluted and plated onto media containing antibiotics (rifampin: 200 mg/l, meropenem: 2 mg/l, and fosfomycin: 16 mg/l) to ensure the absence of bacteria with similar resistance profiles in the intestinal tract. After acclimatization, each mouse was orally challenged with 200 μl of bacterial suspension in PBS solution at a concentration of 10^10^ CFU/ml. The control group received only PBS solution. Fresh fecal samples were collected and weighted daily starting from the following day. Following homogenization of 0.1 g feces, the resulting homogeneous liquid was inoculated onto agar plates containing antibiotics (rifampin: 200 mg/l, meropenem: 2 mg/l, and fosfomycin: 16 mg/l). A minimum detection limit of 1000-CFU/g feces was set to ensure accurate quantification of bacterial populations.

### Statistical analyses

Statistical analyses were performed using Prism 8.0 software (GraphPad, San Diego, CA, USA). The data were expressed as mean ± standard deviation, and the *P* values between two groups were calculated by unpaired Student’s test (nonparametric). The differences in *G. mellonella* larvae were compared using the log-rank (Mantel-Cox) test. Statistical significance was set at *P* < .05.

## Results

### Cointegrate plasmid pL53T reduced the fitness of *E. coli* C600

**Figure 1:**
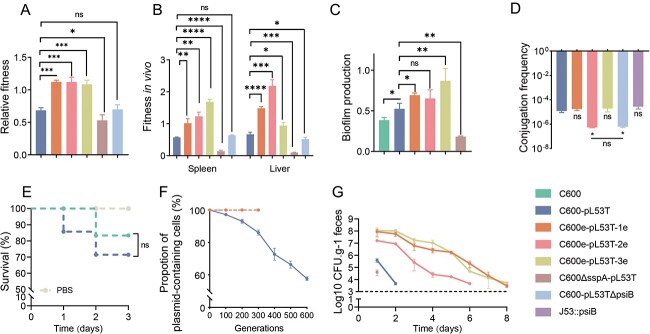
Phenotypic indicators of C600-pL53T during the process of adaptive evolution. Comparison of (A) *in vitro* competitive ability among the ancestral strain, three evolved clones, the *psiB* knockout mutant, and the *sspA* knockout mutant. (B) *In vivo* competitive ability among the ancestral strain, three evolved clones, and the *sspA* knockout mutant. (C) Biofilm formation ability among C600, the ancestral strain, three evolved clones, and the *sspA* knockout mutant. The *y*-axis represents the OD590 nm value. (D) Conjugation frequencies of plasmids in the ancestral strain, three evolved clones, the *psiB* knockout mutant, and the *psiB* complementary strain. The *psiB* complementation was achieved by introducing the *psiB*-carrying plasmid to the recipient strain J53, and then the complementary strain was conjugated with the donor strain C600e-pL53T-2e. (E) Survival rate of *Gelleria mellonella* larvae infected with C600 and C600-pL53T. (F) Plasmid stability among the ancestral strain, and three evolved clones. (G) Murine-gut colonization of the ancestral strain, three evolved clones, and the *sspA* knockout mutant. The log-rank (mantel-cox) test was employed for statistical comparison of the survival rate in *G. mellonella* larvae. Student’s *t*-tests were utilized for other data analysis. ns: nonsignificant, ^*^: *P* < .05, ^**^: *P* < .01, ^***^: *P* < .001, ^****^: *P* < .0001. The tested strains in the figure correspond to a unique color to facilitate comprehension.

To evaluate the fitness cost of pL53T on C600, we initially performed *in vivo* and *in vitro* competition assay between C600-pL53T and the plasmid-free ancestral strain C600. We also compared the virulence and biofilm formation ability of C600-pL53T with those of the plasmid-free ancestral strain C600. The plasmid pL53T impede C600 competitiveness both *in vivo* and *in vitro*. The 24-h *in vitro* RF was determined to be 0.68 ± 0.03 (Fig. 1A). Furthermore, the ratio of drug-resistant bacteria to drug-susceptible bacteria decreased following a 24-h competition *in vivo* mouse model (the ratio of drug-resistant bacteria to drug-susceptible bacteria in the spleen was reduced to 0.572 ± 0.01 times the initial value, whereas the corresponding value for liver was reduced to 0.668 ± 0.05) (Fig. 1B). Moreover, the plasmid did not exhibit any impact on virulence of C600 (log-rank test; *P* = .3532) (Fig. 1E), whereas it significantly augmented the biofilm formation ability of C600 (*P* < .05) (Fig. 1C).

### Changes of phenotypic parameters of C600-pL53T after serial passages under antibiotics selection

The stability of plasmids facilitates their rapid dissemination and expansion into new ecological niches [34]. Therefore, we performed three independent serial passages for about 600 generations in antibiotic-free LB broth at 37°C to assess the plasmid stability of pL53T in C600. The results indicated that the plasmid cannot be maintained stably in the absence of antibiotics, as the proportion of cells carrying the plasmid decreased from 100% at initiation to 57.6% on the 600th generations (Fig. 1F). It was observed that plasmid-encoded *fosA* and *bla*_NDM_ conferred resistance in host bacteria against fosfomycin (MIC = 128 mg/ml) and meropenem (MIC = 32 mg/ml) (Table S2). Therefore, to simulate bacterial evolution in the presence of antibiotic residues, C600-pL53T was subjected to serial passages in triplicate for 600 generations under the subinhibitory concentration of fosfomycin (16 mg/ml) and meropenem (2 mg/ml)-containing broth.

After the passage, we streaked final generation of three parallel groups onto antibiotic-free LB agar plates and randomly selected three endpoint evolved clones, namely, C600e-pL53T-1e, C600e-pL53T-2e, and C600e-pL53T-3e, and conducted a comprehensive examination of various phenotypes both pre- and post-evolution. The fitness of three evolved clones was significantly enhanced as demonstrated by an *in vitro* competition test, with RF of 1.12 ± 0.02, 1.11 ± 0.06, and 1.08 ± 0.05 for C600e-pL53T-1e, C600e-pL53T-2e, and C600e-pL53T-3e, respectively (Fig. 1A). In addition, similar results were observed in an *in vivo* competition assay (Fig. 1B), suggesting that adaptive evolution may compensate for the fitness cost of plasmids during antibiotic selection. Furthermore, the stability of plasmids was obviously increased in three evolved clones, as evidenced by a constant ratio of plasmid-carrying bacteria in the population remaining at 100% after 300 generations of serial passage (Fig. 1F).

Other notable indicators underwent changes as well. In C600e-pL53T-2e, the conjugation frequency of evolved plasmid appeared to have significantly decreased (*P* < .05), whereas in the remaining two strains, the plasmid conjugation frequency was comparable to that of the ancestral plasmid (Fig. 1D). Furthermore, biofilm-forming capacity was significantly enhanced in two of the evolved clones (C600e-pL53T-1e: *P* < .05; C600e-pL53T-3e: *P* < .01), whereas the third clone showed a trend toward improvement (C600e-pL53T-2e: *P* = .1589) (Fig. 1C). Additionally, all three evolved clones exhibited improved gut colonization ability compared with the ancestral strain. The cell counts of the ancestral strain rapidly decline in the gut colonization assay, and became undetectable after only 2 days in the gut colonization assay, whereas the evolved clones were able to survive for up to 6–8 days, highlighting their superior survivability (Fig. 1G).

### Large fragment deletions in pL53T-2e were responsible for the decreased conjugation frequency and improved fitness

**Figure 2:**
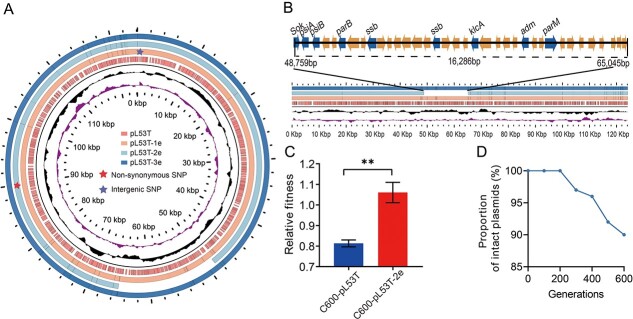
A strategy for plasmid evolution. (A) Mutations and fragment deletions observed in the plasmids of three evolved clones. (B) the detailed structure of the deleted fragment was displayed, with emphasis on the major functional genes. (C) RF of C600-pL53T and C600-pL53T-2e. (D) Dynamic deletion changes of the 16-kb sequence. Statistical comparison was done by Student’s *t*-tests. ^**^: *P* < .01.

WGS was employed to gain deeper insights into the phenotypic changes of evolved clones, with a focus on identifying any gene deletions or additions in the three endpoint evolved clones. We initially mapped the sequencing reads from the evolved clones to the ancestral plasmid. Although only an SNP and a non-synonymous SNP were identified in plasmids pL53T-1e and pL53T-2e, we observed a significant loss of ~16 kb in pL53T-2e (Fig. 2A). The deleted region, which mainly contained *psiA*, *psiB*, *parB*, *parM*, and *klcA* gene (Fig. 2B), may result in a lower conjugation frequency and fitness cost for pL53T-2e. To test the hypothesis, we introduced the evolved plasmid into the ancestral *E. coli* strain C600 and performed a competition assay with an isogenic plasmid-free strain. The RF of ancestral C600 carrying pL53T-2e was substantially higher than that of C600-pL53T (Fig. 2C), suggesting that the evolved plasmid with a structural deletion indeed reduced its fitness cost.

We delved deeper into the issue of altered conjugation frequency. Generally, plasmid-mediated horizontal transfer can trigger an SOS response in recipient cells, and the *psiB* (plasmid SOS interference/inhibition) gene can suppress unwarranted SOS induction during conjugation [35]. Therefore, we constructed a mutant plasmid pL53TΔpsiB and performed a conjugation assay. The results showed that the conjugation frequency of pL53TΔpsiB decreased to a level comparable to that of pL53T-2e (compared with pL53T: *P* < .05; compared with pL53T: n.s.). Complementation of the recipient strain with *psiB* gene restored the conjugation frequency to that of the wild-type plasmid, demonstrating that the absence of the *psiB* gene in pL53T-2e was a significant contributing factor to impaired plasmid transferability (Fig. 1D). Nonetheless, the deletion of *psiB* was found to have no discernible impact on the plasmid fitness cost (Fig. 1A and B), suggesting that *psiB* solely contributes to the alteration of plasmid conjugation transfer phenotype rather than affecting the plasmid fitness cost. To investigate the potential correlation between the improvement of plasmid fitness cost and plasmid copy number, we measured the plasmid copy number and MICs. It was observed that there was no significant change in either the plasmid copy number or MICs before and after evolution (Table S2), indicating that changes in plasmid fitness cost were independent of plasmid copy number. These findings revealed that although sequence deletion events can minimize the fitness cost of plasmids to host bacteria, they also weaken the horizontal transferability. The molecular mechanism underlying the reduced plasmid fitness cost, however, still needs to be elucidated.

As the structural alteration in plasmid pL53T-2e was detected in the last generation of bacterial population, the dynamics of deletion during adaptive evolution remain uncertain. To elucidate these results, we designed primers targeting the partial loss region and analyzed changes in plasmid structure among 96 monoclonal populations sampled every 100 generations from an evolved bacterial population. The frequency of the sequence in the population declined progressively after the 300th generations, with the deletion rate of 3%, 4%, 8%, and 10% for the 300th, 400th, 500th, and 600th, respectively (Fig. 2D).

### Five conserved SNPs were found on the chromosomes of three evolved clones

**Figure 3:**
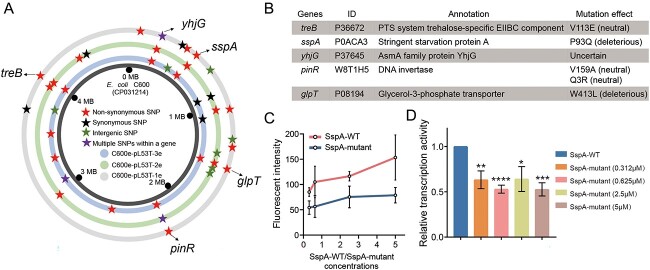
Chromosomal SNPs analysis of the three evolved clones and functional verification of *sspA* mutations. (A) Different SNPs between the three evolved clones and the ancestral strain. (B) Prediction of the effect of single-base mutation on protein function. (C) Transcription activities of the wild-type SspA and its mutant at different concentrations. (D) Relative transcription activities of the wild-type SspA and its mutant at different concentrations. Statistical comparison was done by Student’s *t*-tests. ^*^: *P* < .05, ^**^: *P* < .01, ^***^: *P* < .001, ^****^: *P* < .0001.

Chromosomal mutations, particularly in genes encoding transcriptional regulators, provide insight into the molecular mechanisms underlying bacterial fitness evolution [17]. Consequently, we identified SNPs present on the chromosomes of all evolved clones. Among the three evolved clones, a total of 40 chromosomal loci with mutations were identified, including 24 non-synonymous SNPs, six synonymous SNPs, seven intergenic SNPs, and three multiple SNPs within a gene. Furthermore, five shared non-synonymous SNPs were identified on five genes encoding PTS system trehalose-specific EIIBC component (*treB*), stringent starvation Protein A (*sspA*), asmA family protein YhjG (*yhjG*), serine recombinase (*pinR*), and glycerol-3-phosphate transporter (*glpT*), respectively (Fig. 3A). The gene *treB* is involved in trehalose transport at low osmolarity, which acts an essential role in trehalose uptake [36]. The *pinR* gene is responsible for mediating DNA integration and DNA recombination, whereas the function of *yhjG* gene remains incompletely elucidated with respect to its association with a cellular component. Function prediction through Phyre2 [29] has revealed that SNPs in *glpT* and *sspA* genes may have impact on the structures and functions of their corresponding proteins (Fig. 3B). The *glpT* gene encodes a transport protein that mediates the absorption of sn-glycerol-3-phosphate [37]. The *sspA* gene encodes an RNAP-associated protein that has been demonstrated to modulate bacterial virulence [38] and quorum sensing [39]. Furthermore, a recent study has demonstrated that *sspA* gene plays a positive role in regulating biofilm formation in *Pseudoalteromonas* sp. R3 [40], suggesting that mutation in this gene during adaptive evolution may be responsible for the observed phenotypic changes.

### Mutation of *sspA* played an essential role in the regulation of fitness, biofilm formation, and gut colonization of *E. coli* C600

To confirm the involvement of *sspA* gene in bacterial fitness regulation, we constructed a *sspA* knockout mutant C600ΔsspA-pL53 and conducted both *in vitro* and *in vivo* competition assays. The results demonstrated that the absence of *sspA* compromised the competitiveness of C600-pL53T (Fig. 1A and B). In addition, we have observed that the presence of *sspA* impacts the biofilm formation and gut colonization abilities of C600-pL53T strain. Both these properties were found to be reduced upon loss of *sspA* gene (Fig. 1C and G), indicating an indispensable role played by *sspA* in regulating these phenotypes.

As the SspA protein functions as a transcriptional repressor by binding to the *E. coli* σ70-RNAP holoenzyme as a homodimer [30], we aimed to investigate the impact of *sspA* mutation on evolved clones through a fluorescence-based *in vitro* transcription assay. As SspA activates the *gadA* promoter, its inhibitory capacity is expected to be positively correlated with the transcriptional effect. The results indicate that the mutant exhibits a weakened transcriptional inhibitory ability compared with the wild type, as evidenced by its lower transcriptional activity at different concentrations (Fig. 3C and D). Consequently, the mutation of *sspA* diminishes its capacity for transcriptional inhibition, thereby impacting the modification of diverse phenotypes (improved fitness and reinforced biofilm formation and gut colonization ability) in the three evolved clones.

### Transcriptome analysis revealed that the mutated *sspA* gene facilitated biofilm yield and gut colonization by enhancing flagellar biosynthesis

**Figure 4:**
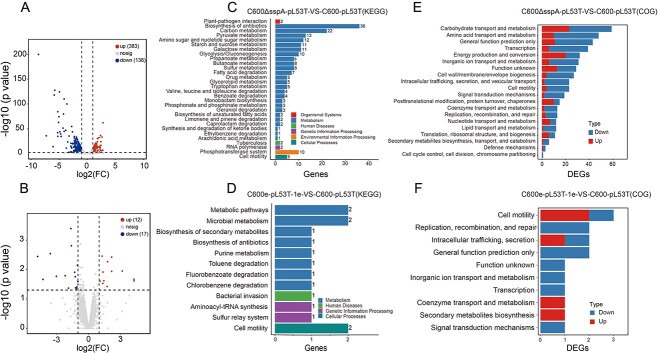
Transcriptomic analysis of the *sspA* knockout mutant, the ancestral strain, and one of the evolved clones C600e-pL53T-1e. (A) Volcano plot of the *sspA* knockout mutant compared with the ancestral strain. (B) Volcano plot of C600e-pL53T-1e compared with the ancestral strain. (C) KEGG enrichment analysis of the *sspA* knockout mutant compared with the ancestral strain. (D) KEGG enrichment analysis of C600e-pL53T-1e compared with the ancestral strain. (E) COG analysis of the *sspA* knockout mutant compared with the ancestral strain. (F) COG analysis of C600e-pL53T-1e compared with the ancestral strain.

To get further insight into the global regulation of the *sspA* gene, we conducted transcriptomic analyses on C600ΔsspA-pL53T, C600-pL53T, and C600e-pL53T-1e. The comparison between C600ΔsspA-pL53T and C600-pL53T revealed the upregulation of 383 genes and downregulation of 136 genes (Fig. 4A). These DEGs were involved in various biological processes, including bacterial metabolism, genetic information processing, environmental information processing, cellular processing, and organismal systems (Fig. 4C). In addition, C600e-pL53T-1e exhibited upregulation of 12 genes and downregulation of 17 genes in comparison to C600-pL53T (Fig. 4B). Considering the diametrically opposite biofilm formation ability, fitness, and gut colonization ability between C600ΔsspA-pL53T and C600e-pL53T-1e, we especially investigated the pathways that could potentially impact these bacterial properties. As expected, DEGs in both data sets were found to be implicated in bacterial motility (Fig. 4C and D). Furthermore, COG analysis indicated that more genes related to cell motility were downregulated in C600ΔsspA-pL53T compared with C600e-pL53T-1e (Fig. 4E and F), which may account for the underlying mechanism responsible for the improved fitness, biofilm formation ability, and gut colonization ability of the evolved clones.

**Figure 5:**
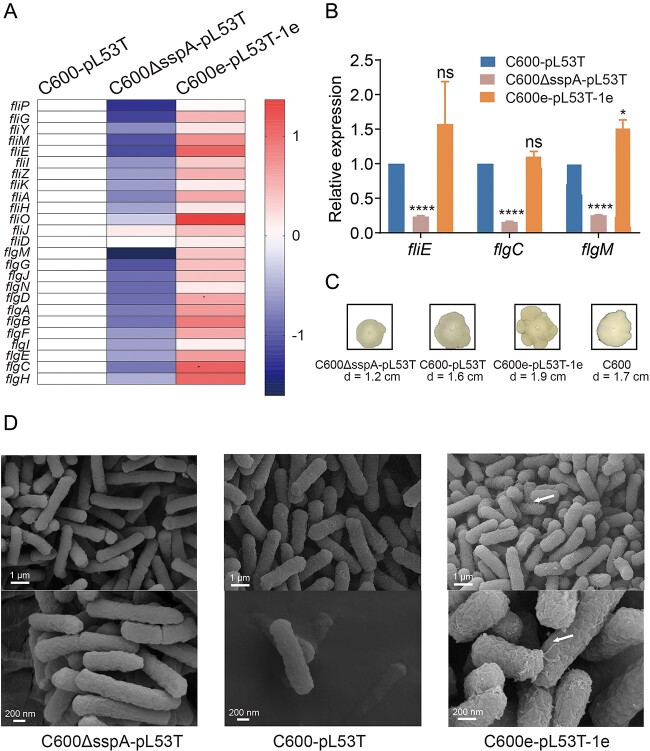
Validation of the transcriptomic results. (A) Relative expression levels of genes related to flagellar assembly in the *sspA* knockout mutant and C600e-pL53T-1e. (B) RT-PCR results of flagella assembly gene *fliE*, *flgC*, and *flgM*. (C) Motility of the *sspA* knockout mutant, the ancestral strain, the *sspA* knockout mutant, and C600e-pL53T-1e. (D) Scanning electron microscope results of the *sspA* knockout mutant, the ancestral strain, and C600e-pL53T-1e. The white arrows represent the flagellar structures. Statistical comparison was done by Student’s *t*-tests. ns: nonsignificant, ^*^: *P* < .05, ^****^: *P* < .0001.

To validate the hypothesis, a comparison was made between the expression levels of genes related to flagellar assembly in C600ΔsspA-pL53T and C600e-pL53T-1. The expression in all selected genes associated with flagellar assembly was upregulated in C600e-pL53T-1e, whereas a reverse result was observed in C600ΔsspA-pL53T (Fig. 5A), which was further confirmed by RT-PCR analysis (Fig. 5B). Consequently, the motility of C600, C600ΔsspA-pL53T, C600-pL53T, and C600e-pL53T-1e was compared. The results demonstrated that the motility of C600 and C600-pL53T were comparable, whereas the motility of C600e-pL53T-1e was higher than that of C600ΔsspA-pL53T, as indicated by their respective secretory diameters measuring 1.9 and 1.2 cm, suggesting the mutation in *sspA* enhanced the bacterial motility during adaptive evolution (Fig. 5C). Moreover, distinct morphological differences were observed between C600e-pL53T-1e and C600ΔsspA-pL53T through scanning electron microscopy. The surface of C600ΔsspA-pL53T appeared flat and smooth without any discernible flagellar structure, whereas the surface C600e-pL53T-1e was rough and uneven with a conspicuous presence of flagellar structures (Fig. 5D). These data revealed that the mutant SspA protein facilitated biofilm formation and gut colonization ability by augmenting flagellar biosynthesis, thereby improving bacterial fitness.

## Discussion

With the advancement of sequencing technology, the complete structure and formation mechanism of cointegrate plasmids have been well characterized [5, 41]. Despite extensive exploration into their structural properties, less is known about the factors contributing their long-term persistence and spread within a specific bacterial host in certain ecological niches. In fact, understanding the key factors affecting plasmid dissemination is of greater significance than analyzing the structure and formation mechanisms. Our preliminary findings have confirmed that the initial fitness cost imposed by the cointegrate plasmid could be compensated for through serial passages under selective pressure [2]. This phenomenon has also been observed in other types of plasmids, such as the IncHI2 plasmid in *Salmonella* Typhimurium [16], and IncR or IncC in *E. coli* [42]. Herein, we introduced the cointegrate plasmid pL53T into *E. coli* C600, followed by long-term evolution to study the mutations or structural alterations.

Our study differs from previous ones in that we have identified compensatory mutations occurring not only in chromosomes but also in plasmids [1, 42-44], including deletions of the latter and mutations affecting chromosomal transcriptional regulatory genes, suggesting the diversity of evolutionary routes in diverse host-plasmid combinations. As predicted by an individual-based model from another study [45], the fate of plasmids within bacterial populations can be ultimately summarized as follows: plasmid loss, acquisition of plasmid accessory genes by bacterial chromosomes, and activation of compensatory evolution. The plasmid-borne genes confer benefits to bacteria, but they usually impose an additional burden on the host cells [43]. Plasmid will take a number of countermeasures to avoid being abandoned by the host bacteria. The stability of plasmid pL4–3, for example, can be enhanced by acquiring a TA system from an endogenous bacterial plasmid [46], whereas IncHI2 plasmid pJXP9 can offset its cost by eliminating MDR regions and conjugation transfer regions [16]. Our study has also identified another strategy through the detection of a large fragment deletion in an evolved clone. This fragment, which has been demonstrated to be widely conserved among conjugative plasmids [47, 48], was located on the IncFII(pCoo) plasmid, and its absence resulted in decreased bacterial horizontal transferability and fitness costs. Further investigation revealed that the absence of *psiB* gene was the primary determinant of decreased conjugation frequency. The *psiB* gene is implicated in the inhibition of SOS response triggered by conjugation [35]. Consistent with this finding, a recent study found that plasmid pED208 lacking *psiB* induced significantly stronger SOS response than its counterpart, which may underline the impediment to horizontal transfer of the plasmid [49].

Although the deletion of the *psiB* gene results in a significant reduction in conjugation frequency, the decrease in plasmid cost cannot be attributed to it. A possible explanation is that the expression of *psiB* is influenced by the functionality of promoters on a given conjugative plasmid and cellular circumstances. Evidence supports this notion, as activation of the F plasmid *psiB* promoter appears to occur exclusively during conjugation [50]. Because *psiB* is independent of the plasmid fitness cost reduction, we hypothesized that the loss of genes involved in partitioning may be responsible for the phenomenon. Partitioning proteins have multifunctional roles, and apart from facilitating DNA segregation, the expression of partitioning genes also influences plasmid fitness cost [44]. Because of the presence of *par* genes on both daughter plasmids of the cointegrate plasmid and its high cost, the functional redundancy may result in the loss of partitioning genes. The absence of the partitioning system may potentially impact plasmid conjugation as well. In addition to its well-accepted role in plasmid vertical transmission [51], the proteins involving in the partitioning system have been found to interact with proteins from the type IV secretion system, thereby facilitating the assembly of the conjugation machinery [52]. However, we anticipate that this effect might not be as significant as the loss of *psiB* because of a potential compensatory function provided by partitioning genes on another daughter plasmid. Hence, compared with the dissociated state of two daughter plasmids, the potential selection of fusion plasmids during the evolutionary process may exhibit greater diversity because of the overlap of some gene functions. Furthermore, the plasmid evolutionary pathway observed in this study may be more conductive to vertical rather than horizontal transmission, thereby rendering cross-species transmission improbable in future. This opinion is substantiated by two factors: first, we did not find any relevant reports or wild-type strains with similar fragment deletion on IncFII plasmids, indicating that such deletion event may occur in specific host bacteria or under certain conditions. Moreover, the rate of deletion did not exhibit a significant increase over time. Second, recent research has investigated two plasmids, pKSR100 and pAPR100, which circulated in the same network but exhibited distinct epidemiological characteristics. Plasmid pKSR100 displayed superior conjugation ability compared with the less prevalent plasmid pAPR100; however, bacteria carrying pKSR100 displayed a reduced SOS response in the presence of antimicrobials. Comparative genomic analysis revealed that pKSR100 contained a gene cluster consisting of five genes, including the *psiB* gene, which could account for the discrepancy in prevalence between the two plasmids [53].

A mutation in the *sspA* gene located on chromosome of all evolved clones represents an additional mechanism for enhanced fitness. The *sspA* gene is the first to be implicated in bacterial fitness and functions as a transcriptional repressor for regulating transcription. The SspA protein specifically binds to the *E. coli* σ^70^-RNAP holoenzyme and interacts with σ^70^ Region 4 and zinc binding domain of β′ subunit, thereby inhibiting transcription initiation by suppressing promoter escape [30]. SspA has been demonstrated to modulate bacterial virulence [38], the quorum sensing system [39], and acid tolerance [54]. To comprehensively address the role of SspA in bacterial fitness regulation, we aim to discuss the potential concerns that may arise among readers. First, because the evolved clone with *sspA* mutation harbors additional genetic mutations, whether other gene mutations interfere with the improvement of bacterial fitness. According to preliminary findings, compensatory mutations in global regulatory proteins are a mainstream evolutionary trajectory by which bacterial hosts can counteract the cost imposed by horizontally acquired DNA [46, 55, 56]. The outcome led us to shift our attention toward the identification of mutations in transcriptional regulators within the evolved clones. Upon comparison with the ancestral strain, only six genes were found to have missense mutations in C600e-pL53T-1e, which may account for the limited number of DEGs observed in transcriptome analysis (Table S3). Moreover, all the three parallel evolved clones showed improved fitness, and the only shared gene with missense mutations affecting transcriptional regulation was *sspA* gene. Therefore, it is reasonable to conclude that the *sspA* gene plays a dominant role in regulating bacterial fitness. Further investigation is needed to determine whether mutations in other genes affect bacterial fitness. Second, What is the underlying mechanism by which the mutated SspA affects bacterial flagellar synthesis? There are three classes of promoters responsible for the transcription of the flagellar operons, among which both Class I promoter and Class II promoter are transcribed by σ^70^ RNAP [57, 58]. σ^70^ RNAP is the sole target of SspA binding [30], implying *sspA* mutations may facilitate flagellar assembly by influencing Class I and Class II promoters of the flagellar operon. Third, as a transcriptional repressor, the reason why *sspA* knockout mutant exhibits decreased flagellar synthesis and biofilm formation abilities. This may be attributed to the substation of SspA’s function by other transcriptional regulators, as evidenced by significant changes in the transcription levels of certain transcriptional regulators observed in *sspA* knockout mutant from transcriptomics analyses. In addition, previous research has found that a significant reduction in biofilm formation in the *sspA* knockout mutant compared with the wild-type strain [40], which is consistent with our findings. Fourth, Whether the flagellar biosynthesis is related to bacterial fitness. A myriad of studies has demonstrated the indispensable role of flagella in bacterial biofilm and gut colonization ability. Flagella’s primary function in the initial stage of biofilm formation is to facilitate motility, thereby promoting cell-to-surface adhesion by overcoming repulsive forces [59]. For gut colonization, flagellum-mediated motility assists bacteria in entering the epithelium via the intestinal mucus layer, enabling bacteria to scan epithelial cells for permissive entry sites [60]. Furthermore, flagella-deficient uropathogenic *E. coli* was outcompeted by wild-type strain in the urinary tract, indicating that flagella contribute to bacterial fitness [61]. Finally, whether the *sspA* mutation is specifically adapted to the medium conditions or influenced by the presence of the plasmid. We think that mutations in *sspA* may be adapted to both. The reasons are as follows: First, the previous study focused on the adaptation of *E. coli* to long-term serial passage in LB broth and observed a parallel occurrence of *sspA* mutation in passaged populations. Furthermore, the *sspA* mutant exhibited superior competitive ability compared with the wild-type parent strain, which aligns with our findings. However, the underlying mechanisms involved were not addressed in their research [62]. This available evidence suggests that mutations in the *sspA* gene for adapting to medium conditions are common and can substantially improve bacteria fitness. Second, the function of the *sspA* gene primarily involves regulating the expression level of H-NS [54], a key factor influencing the fitness cost of IncX3 plasmid [63]. In light of the widespread presence of H-NS on this plasmid, the *sspA* mutation may potentially adapt to the plasmid by reducing the fitness cost through modulation of H-NS expression. This could be another way of regulating plasmid in addition to the bacterial flagellar assembly pathway, which warrants further investigation.

Unlike most previous studies that investigated evolutionary dynamics using *in vitro* evolutionary models [64, 65], this study evaluated the competitiveness and gut colonization ability of evolved plasmid-carrying bacteria *in vivo*, thereby highlighting the likelihood of transmission from environmental sources to mammals. Although the overall fitness effects of plasmids measured *in vitro* were consistent with those measured in mouse models [66], other experimental models, such as *Caenorhabditis elegans*, *G. mellonella*, and mice or pigs, can still be utilized to objectively assess the fitness of plasmid-carrying bacteria within clinically relevant models [12].

This study has revealed that the adaptive evolution to antibiotic exposure can ameliorate the high fitness cost of *bla*_NDM_-bearing cointegrate plasmid-carrying strains, and this evolution process is jointly driven by both chromosome and plasmid. As a result, the risk of persistence and further transmission of the cointegrate plasmid will inevitably be aggravated. Nevertheless, considering the diversity of cointegrate plasmids and the complexity of evolutionary dynamics under different conditions, further studies are still warranted to explore universal targets that specifically impact the spread of cointegrate plasmids.

## Author contributions

Ruichao Li and Zhiqiang Wang conceived and supervised this study. Ziyi Liu designed the experiments and drafted the manuscript. Ziyi Liu, Yanyun Gao, and Fulin Wang performed the experiments. Mianzhi Wang and Yuan Liu analyzed the data. Ruichao Li, Jing Shi, and Ziyi Liu revised the manuscript. All authors approved the final draft.

## Conflicts of interest

None declared.

## Funding

This study was financially supported by the National Natural Science Foundation of China (32161133005 and 32373061), the Jiangsu Agricultural Science and Technology Innovation Fund (CX(21)2010), the China Postdoctoral Science Foundation (2022T150555), and the Priority Academic Program Development of Jiangsu Higher Education Institutions (PAPD).

## Data availability

The RNA sequencing data in this study have been deposited in the NCBI SRA database (BioProject: PRJNA949393). The draft sequences of the evolved clones and the ancestral strain have been deposited in the NCBI GenBank database (BioProject: PRJNA949418). Other data that support the findings of this research are available upon request.

## Supplementary Material

Supplementary_data_revised_wrae037
