# Supplementary material for: Adaptive evolution of plasmid and chromosome contributes to the fitness of a blaNDM-bearing cointegrate plasmid in Escherichia coli
Source: ISME J. 2024 Mar 4;18(1):wrae037. doi: 10.1093/ismejo/wrae037 (PMC10976473; doi:10.1093/ismejo/wrae037)
Supplement: Supplementary_data_revised_wrae037 [file supplementary_data_revised_wrae037.docx]

**Supplemental information**

**Supplemental methods**

**Preparation of SspA and its mutant protein, *E. coli*** **σ^70^ protein, and *E. coli* RNAP core enzyme**

According to a previous study (1), the *E. coli sspA* gene or its mutant gene was cloned into pET28a, resulting in the plasmid pET28a-NH-SspA encoding N hexahistidine-tagged SspA protein. The recombinant plasmid was further transformed into *E. coli* strain BL21(DE3), and a single clone was selected for inoculation into 1L of LB broth supplemented with 50 μg/mL kanamycin. The culture was incubated at 37℃ until the OD600 reached 0.6, at which point induction of SspA protein expression was achieved by adding 0.5 mM isopropyl-β-D-thiogalactoside (IPTG) to the medium. Following overnight incubation at 20℃, the cultures were harvested and resuspended in 20 mL of buffer A. Subsequently, cell lysis was performed using an ATS AH-10013 cell disrupter (ATS, Inc.), followed by centrifugation of the lysate at 4℃ and 12000 rpm for a duration of 45 min. The supernatant was then transferred into a 2 mL Ni-NTA agarose column equilibrated with buffer A. Subsequently, the column was washed with 10 mL of buffer A containing 0.16 M imidazole and eluted with 10 mL buffer A containing 0.5 M imidazole. The eluted sample underwent purification by anion-exchange chromatography on a Mono Q 10/100 GL column (GE Healthcare, Inc.). The purified protein was stored at -80℃. Meanwhile, a BCA protein assay kit (Pierce™ BCA Protein Assay Kit, Thermo Scientific™, Inc.) was used to determine the protein concentration.

The *E. coli* σ^70^ protein and RNAP core enzyme were prepared using plasmid pGEMD and *E. coli* strain BL21(DE3) transformed with plasmid pIA900, respectively, as reported (2).

**Plasmid copy number measurement**

According to the previous study (3), three independent DNA extractions using TIANamp bacterial DNA kit (TianGen, Beijing, China) were performed for ancestral and evolved strains. qPCRs targeting the IncX3 replicon and monocopy chromosomal gene *uidA* were carried out in triplicate for each extraction (4). Plasmid copy number per chromosome was calculated using the formula according to a previously published method (5). Cn=[(1+Ec)^Ctc^/(1+Ep)^Ctp^]×(Sc/Sp). The plasmid copy number per chromosome (Cn) is determined by the sizes of the chromosomal and plasmid amplicons (Sc and Sp, respectively), the efficiencies of the chromosomal and plasmid qPCRs (Ec and Ep, relative to 1), as well as the threshold cycles of the chromosome and plasmid reactions Ctc and Ctp, respectively.

**Antimicrobial susceptibility testing (AST)**

The AST of ancestral and evolved strains against meropenem was performed based on the broth microdilution method, and interpreted according to CLSI guidelines (6). For fosfomycin, we employed agar dilution to determine the MICs as previously described (7).

**Analysis of *psiB*-positive plasmids based on PLSDB database**

To investigate the distribution of *psiB*-carrying plasmids among different species and plasmid types, a BLASTn search was conducted on all available *psiB*-carrying plasmids based on PLSDB database as of June 1, 2022) (8). The retrieved plasmids were then classified bases on various parameters (bacterial species, geographical distribution, temporal distribution, length, GC contents, and plasmid types), and counted accordingly.

**Supplemental text**

To investigate the distribution pattern of *psiB*-bearing plasmids from various perspectives. A total of 437 *psiB*-bearing plasmids were retrieved from the PLSDB database as of June 1, 2022. From a geographical perspective, the *psiB* positive plasmids were primarily distributed in China, the United States, India, and Europe. However, they have also been sporadically detected in Oceania and select countries within the Middle East (Figure S1a). From the perspective of discovery time, the *psiB* positive plasmids were mainly identified post-2016 (Figure S1b). In addition, these plasmids exhibited a median length and GC content of 104,623 bp and 52.42%, respectively (Figure S1c,d). Notably, the distribution of these plasmids may be influenced by both host and plasmid type, as they displayed a strong correlation with IncF plasmid, and are exclusively found in Enterobacterales, with *E. coli* and *K. pneumoniae* being the primary host bacteria (Figure S1e,f).

**Supplemental tables**

**Table S1. Primer sequences involved in this study.**

|  | Genes | Sequences | Length/bp |
| --- | --- | --- | --- |
| Verification of transcriptome results | 16S rRNA gene | F: CCTACGGGAGGCAGCAG | 194 |
|  |  | R: GCATCGCTTTTTCTGTCGCT |  |
|  | *fliE* | F: CCGCAACCGACCATTAGTT | 111 |
|  |  | R: GGGTTCACCGAGAGTGAATTT |  |
|  | *flgC* | F: CCCGGACAAACTGGTTTATGA | 92 |
|  |  | R: CCATCTCTCCGACAACATCAA |  |
|  | *flgM* | F: AGCACCAGTGTGACGTTAAG | 88 |
|  |  | R: CCGTTACGAATCGCCAGTT |  |
|  | *fliO* | F: ATTAGCGCCAGTGCTTCA | 100 |
|  |  | R: GATTGATTTGACCTGCGGTAAC |  |
|  | *flgH* | F: GATACTGTGCCGCGCTATTT | 124 |
|  |  | R: ACGTGCCGCTAAAGGTATTG |  |
| Confirmation of the cointegrate plasmid | Fusion site 1 | F: ACGGATCGGACAGGTTGAGA | 1803 |
|  |  | R: CGCCTCGCCACACTAAT |  |
|  | Fusion site 2 | F: CTTGGTCTGACAGTTAC | 2097 |
|  |  | R: TTCACGGTCAGGGGTGGTCG |  |
|  | IncX3 replication gene | F: GAGGCTTATCGTGAAGACAGTAA  R: CAGAGCTGCATAAGAGGCATA |  |
| Verification of the partial loss region | Lose region | F: CATACGGATATTCCGCCAGC | 3031 |
|  |  | R: CCCGTTCTGACTGGCCTGACTGT |  |
| Knockout of *psiB* | Introduction of N20 | GAGTATGGCAGCGAGTTCGGGTTTTAGAGCTAGAAATAGCAAGTTAAAATAAG |  |
|  |  | CCGAACTCGCTGCCATACTCATGGAGAAACAGTAGAGAGTTGC |  |
|  | Construction and introduction of homologous arms | HA1-F: aaacaaataggggttccgcgCACGGGCTTTCTGCTCTG | 516 |
|  |  | HA1-R: ATGAGCGCACGGGGTACAGCGTCAGTGA |  |
|  |  | HA2-F: GCTGTACCCCGTGCGCTCATGGTCTGTA | 514 |
|  |  | HA2-R: tctccttacgcatctgtgcTCCCTGCTCAGTTTCTGC |  |
|  | Identification of knockouts | F: GCTCACCCCACTGCCAGAACA | Failure: 1573  Success:1138 |
|  |  | R: TGACCAGCGATGCGCCAT |  |
| Knockout of *sspA* | Introduction of N20 | AATGATGCGAGATTCCCACAGTTTTAGAGCTAGAAATAGCAAGTTAAAATAAG |  |
|  |  | TGTGGGAATCTCGCATCATTATGGAGAAACAGTAGAGAGTTGC |  |
|  | Construction and introduction of homologous arms | HA1-F: aaacaaataggggttccgcgGTCCCTGCGTTCTGAACT | 543 |
|  |  | HA1-R: GACAAATCCATGAAAACCTCCAGGTATAGTCAGAA |  |
|  |  | HA2-F: CTGGAGGTTTTCATGGATTTGTCACAGCTAACACC | 527 |
|  |  | HA2-R: tctccttacgcatctgtgcACTTCACAACGCGTAATGC |  |
|  | Identification of knockouts | F: CAGGTTGAACGTAAGAAAGT | Failure: 1559  Success:920 |
|  |  | R: TGCGGAGGTTCATCGTCAG |  |
| Plasmid copy number measurement | *uidA* | GTCAATAATCAGGAAGTG | 201 |
|  |  | AAAGAAATCATGGAAGTAA |  |
|  | IncX3 | CGTGAAGACAGTAACGGGATTA | 76 |
|  |  | AAGGGAAAGGGATGCAAGAG |  |

**Table S2. Plasmid copy number, and MICs of ancestral and evolved strains.**

| Strains | Plasmid copy number | MICs (mg/L) of meropenem | MICs (mg/L) of fosfomycin |
| --- | --- | --- | --- |
| C600-pL53T | 0.25±0.03 | 32 | 128 |
| C600e-pL53T-1e | 0.24±0.24 | 32 | 128 |
| C600e-pL53T-2e | 0.23±0.01 | 32 | 128 |
| C600e-pL53T-3e | 0.21±0.02 | 32 | 128 |

**Table S3. SNPs identified in three evolved strains as compared with the ancestral strain.**

| Strain | SNP (amino acid substitution) | Nucleotide position | Gene or Product |
| --- | --- | --- | --- |
| C600-pL53T-1e | C→A (-) | 309/981 | *pilT* |
|  | C→A (Pro93Gln) | 278/639 | *sspA* |
|  | G→T (Glu156*) | 466/633 | *dnaL* |
|  | T→A (-) | 338/1422 | *treB* |
|  | A→C (-)  T→A (-)  A→G (-)  G→A (-)  T→C (-) | 738/1044  783/1044  789/1044  801/1044  810/1044 | yhhI |
|  | G→T (-) | Spacer (17745/Node 53)^1^ |  |
|  | G→T (-) | Spacer (105242/Node 12) |  |
|  | C→A (Lys632Asn) | 1896/3150 | *acrB* |
|  | C→A (Ser324*) | 971/2433 | yaeT |
|  | G→T (-) | Spacer (15643/Node 112387) |  |
|  | T→G (-) | 303/591 | *pinR* |
|  | C→A (Thr307Lys) | 920/2061 | *yhjG* |
|  | G→T (Trp413Leu) | 1238/1359 | *glpT* |
| C600-pL53T-2e | C→A (-) | 291/879 | *xdhB* |
|  | C→A (Pro93Gln) | 278/639 | *sspA* |
|  | G→T (Met615Ile) | 2085/2697 | P-type ATPase |
|  | T→A (-) | 338/1422 | *treB* |
|  | G→A (-) | Spacer (134728/Node 2) |  |
|  | C→A (Ala134Glu) | 401/516 | *ompX* |
|  | T→C (-) | Spacer (268194/Node 2) |  |
|  | G→T (-) | 594/984 | 2-keto-3-deoxygluconate transporter |
|  | G→T (-) | 48/1836 | *virD4* |
|  | C→A (Asp76Met) | 226/1632 | Na/Pi cotransporter family protein |
|  | C→T (-) | Spacer (59760/Node 11) |  |
|  | C→A (Pro172Thr) | 514/768 | Formate hydrogenlyase subunit 7 |
|  | A→G (Gln3Arg)  T→A (-)  T→C (Val159Ala) | 8/591  447/591  476/591 | *pinR* |
|  | C→A (Thr307Lys) | 920/2061 | *yhjG* |
|  | G→T (Trp413Leu) | 1238/1359 | *glpT* |
| C600-pL53T-3e | C→A (Pro93Gln) | 278/639 | *sspA* |
|  | T→A (-) | 338/1422 | *treB* |
|  | T→C (-) | Spacer (268194/Node 2) |  |
|  | C→A (Ser437*) | 1310/1575 | *nikA* |
|  | G→T (-) | Spacer (139563/Node 6) |  |
|  | G→T (-) | 834/2598 | *htrE* |
|  | G→T (Ala207Ser) | 619/1497 | L-xylulose/3-keto-L-gulonate kinase |
|  | T→C (-) | 462/1029 | Translation elongation factor Tu |
|  | G→T (Cys188Phe) | 563/1278 | *yqcE* |
|  | G→T (Gly429Cys) | 1285/1404 | Acetaldehyde dehydrogenase |
|  | A→G (Gln3Arg) | 8/591 | *pinR* |
|  | C→A (Ala10Glu)  C→A (Thr307Lys) | 29/2061  920/2061 | *yhjG* |
|  | G→T (Trp413Leu) | 1238/1359 | *glpT* |

(-), synonymous mutation.

*, including a stop codon.

^1^, intergenic mutation, for example, 17745/Node 53 means a mutation occurred at 17745 loci in Node 53.

**Supplemental figures**

**
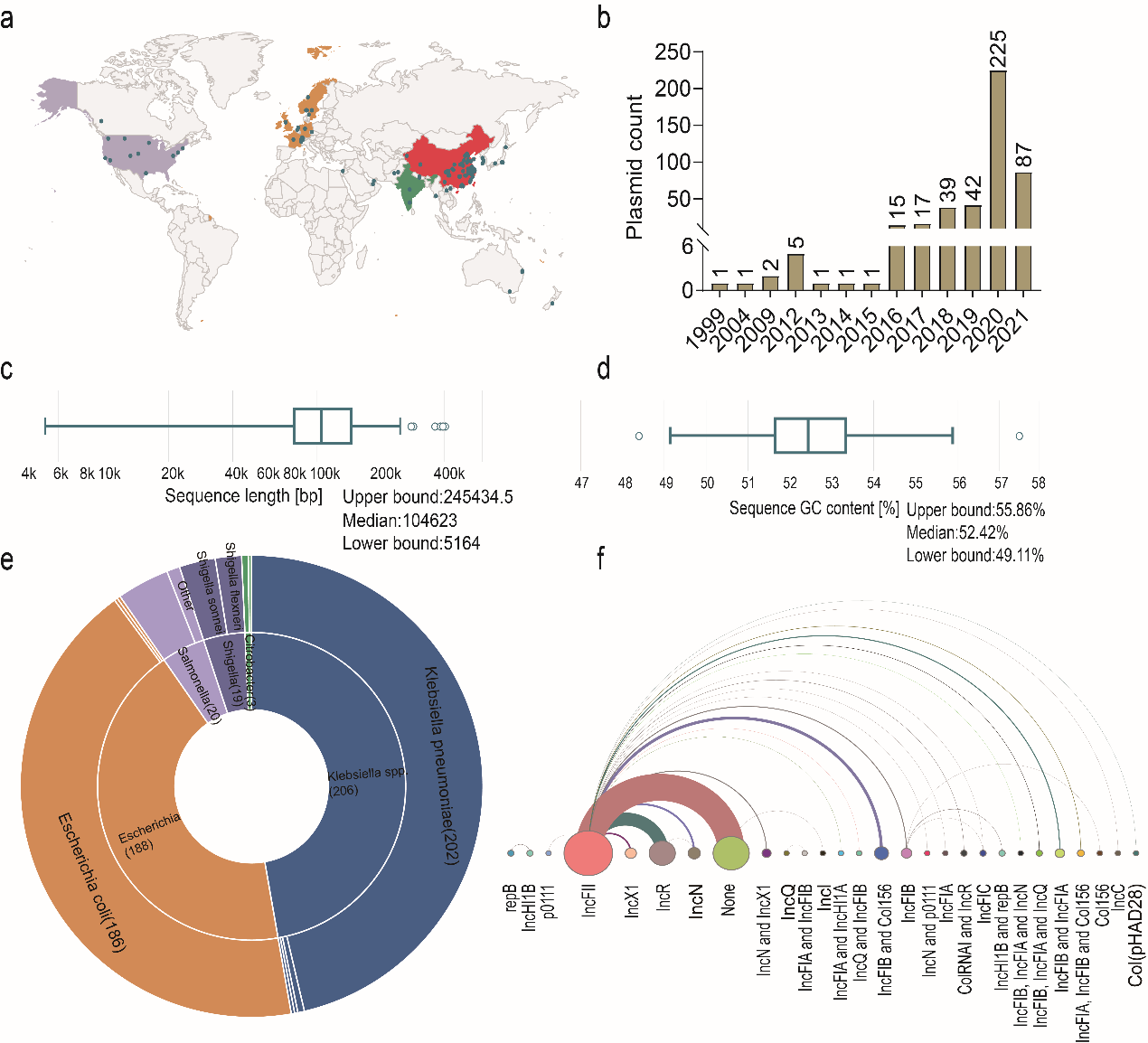
 Figure S1. The distribution of *psiB*-carrying plasmids.** a: Geographical distribution of *psiB* positive plasmids. b: Temporal distribution of *psiB* positive plasmid. c: Length analysis of *psiB* positive plasmids. d: GC content analysis of *psiB* positive plasmids. e: Taxonomy of strains carrying *psiB* positive plasmid. f: Distribution of *psiB* positive plasmids types. Different circles represent different plasmid replicons, and the connection lines represent that the plasmid simultaneously contains the corresponding replicons. The thicker the connection line or the larger the circle, the more plasmids belong to the replicon. The data are obtained from PLSDB database, and plasmids with clear relevant information will be included in the statistical scope, otherwise, they will be excluded.

**References**

1. Wang F, Shi J, He D, Tong B, Zhang C, Wen A, et al. Structural basis for transcription inhibition by *E. coli* SspA. Nucleic Acids Res. 2020;48(17):9931-42.

2. Feng Y, Zhang Y, Ebright RH. Structural basis of transcription activation. Science. 2016;352(6291):1330-3.

3. San Millan A, Heilbron K, MacLean RC. Positive epistasis between co-infecting plasmids promotes plasmid survival in bacterial populations. ISME J. 2014;8(3):601-12.

4. Rozwandowicz M, Brouwer MSM, Mughini-Gras L, Wagenaar JA, Gonzalez-Zorn B, Mevius DJ, et al. Successful Host Adaptation of IncK2 Plasmids. Front Microbiol. 2019;10:2384.

5. San Millan A, Santos-Lopez A, Ortega-Huedo R, Bernabe-Balas C, Kennedy SP, Gonzalez-Zorn B. Small-plasmid-mediated antibiotic resistance is enhanced by increases in plasmid copy number and bacterial fitness. Antimicrob Agents Chemother. 2015;59(6):3335-41.

6. CLSI. Performance standards for antimicrobial susceptibility testing: twenty-fourth informational supplement, M100-S28. CLSI, Wayne, PA. 2018.

7. Van den Bijllaardt W, Schijffelen MJ, Bosboom RW, Cohen Stuart J, Diederen B, Kampinga G, et al. Susceptibility of ESBL *Escherichia coli* and *Klebsiella pneumoniae* to fosfomycin in the Netherlands and comparison of several testing methods including Etest, MIC test strip, Vitek2, Phoenix and disc diffusion. J Antimicrob Chemother. 2018;73(9):2380-7.

8. Galata V, Fehlmann T, Backes C, Keller A. PLSDB: a resource of complete bacterial plasmids. Nucleic Acids Res. 2019;47(D1):D195-D202.
